# Seasonal influenza vaccination of healthcare workers: systematic review of qualitative evidence

**DOI:** 10.1186/s12913-017-2703-4

**Published:** 2017-11-15

**Authors:** Theo Lorenc, David Marshall, Kath Wright, Katy Sutcliffe, Amanda Sowden

**Affiliations:** 1 Centre for Reviews and Dissemination, University of York, York, YO10 5DD UK; 2 Evidence for Policy and Practice Information and Co-ordinating Centre (EPPI-Centre), Social Science Research Unit, UCL Institute of Education, University College London, 18 Woburn Square, London, WC1H 0NR UK

**Keywords:** Healthcare workers, Influenza, Qualitative research, Systematic review, Vaccination

## Abstract

**Background:**

Most countries recommend that healthcare workers (HCWs) are vaccinated seasonally against influenza in order to protect themselves and patients. However, in many cases coverage remains low. A range of strategies have been implemented to increase uptake. Qualitative evidence can help in understanding the context of interventions, including why interventions may fail to achieve the desired effect. This study aimed to synthesise evidence on HCWs’ perceptions and experiences of vaccination for seasonal influenza.

**Methods:**

Systematic review of qualitative evidence. We searched MEDLINE, EMBASE and CINAHL and included English-language studies which reported substantive qualitative data on the vaccination of HCWs for seasonal influenza. Findings were synthesised thematically.

**Results:**

Twenty-five studies were included in the review. HCWs may be motivated to accept vaccination to protect themselves and their patients against infection. However, a range of beliefs may act as barriers to vaccine uptake, including concerns about side-effects, scepticism about vaccine effectiveness, and the belief that influenza is not a serious illness. HCWs value their autonomy and professional responsibility in making decisions about vaccination. The implementation of interventions to promote vaccination uptake may face barriers both from HCWs’ personal beliefs and from the relationships between management and employees within the targeted organisations.

**Conclusions:**

HCWs’ vaccination behaviour needs to be understood in the context of HCWs’ relationships with each other, with management and with patients. Interventions to promote vaccination should take into account both the individual beliefs of targeted HCWs and the organisational context within which they are implemented.

**Electronic supplementary material:**

The online version of this article (10.1186/s12913-017-2703-4) contains supplementary material, which is available to authorized users.

## Background

Most countries recommend that healthcare workers (HCWs), at least those involved in direct patient care, are vaccinated against influenza each winter [1]. Seasonal influenza vaccination can help to protect not only HCWs but also patients against infection. A recent systematic review found that vaccination of HCWs significantly reduced influenza-like illness and all-cause mortality among patients, [2] although results for other outcomes such as number of working days saved are more equivocal [3].

However, many HCWs decline vaccination. Vaccine coverage among HCWs in the USA has surpassed 75%, [4] but in many European countries it remains below 30% [5]. A range of strategies have been implemented to increase vaccination among HCWs. A recent systematic review indicates that mandatory vaccination policies, awareness-raising and interventions to increase the accessibility of vaccination are likely to be effective, but that incentives and education are ineffective [6]. Many quantitative studies have examined HCWs’ attitudes to vaccination and the determinants of vaccination uptake [7]. Qualitative evidence may complement these quantitative data by highlighting potential barriers and facilitators of vaccination uptake, which can then be targeted in future interventions and strategies.

The aim of this systematic review was to synthesise evidence on HCWs’ perceptions and experiences of vaccination for seasonal influenza. It was commissioned by the Department of Health in England to inform the development of policy on vaccination of HCWs. We used a systematic approach, with pre-defined inclusion criteria and a reproducible methodology. However, due to the need to provide a timely synthesis of the evidence for policy decision-making, we streamlined our approach to data analysis, which was conducted by a single reviewer and focused on the identification of key themes, rather than on producing a critical synthesis or developing third-order constructs. Further interpretations of the data are explored in the discussion below.

## Methods

We followed CRD Guidance on Undertaking Systematic Reviews [8].

### Searching

We searched MEDLINE, EMBASE and CINAHL in May–June 2016. The search strategy took the form: (terms for HCWs) AND (terms for vaccination) AND (terms for influenza) AND (terms for views and qualitative research).

The full MEDLINE search strategy is presented in web-only Additional file 1. No date or language restrictions were applied to the search. We searched Google using simplified forms of the search strategy and scanned the first 100 results, and manually searched websites of key organisations including NHS Employers, Centers for Disease Control and Prevention and the World Health Organization. We screened a recently published review evaluating the effectiveness of interventions to increase influenza vaccine uptake for any linked qualitative data [6]. We scanned the lists of included studies of potentially relevant systematic reviews identified by the search, and the reference lists of all included studies. We carried out forward citation chasing on all included studies using Google Scholar.

### Screening

A 10% sample of abstracts was screened by two reviewers independently and differences resolved by discussion. Agreement on inclusion for this sample was 99.4% (κ = 0.66). The remaining 90% were screened by a single reviewer. The following inclusion criteria were applied:

1. Qualitative research
2. Study reports data on seasonal influenza vaccination
3. Study includes HCWs
4. Study published in English
5. (full-text only) Study reports substantive qualitative data (i.e., more than one or two relevant data points, or a very brief summary of findings)

All full-text studies were screened by two reviewers independently and differences resolved by discussion.

### Quality assessment, data extraction and synthesis

We used Hawker et al.’s tool to assess study quality [9]. Quality assessment was carried out by one reviewer and checked in detail by a second reviewer. Data were extracted on the methodology and characteristics of the study, including: research question; sampling and recruitment; study population; data collection; and data analysis. We used a thematic analysis methodology for the synthesis of qualitative data [10]. Thematic qualitative data were coded from the findings of the studies, including quotes from participants and study authors’ interpretations. The themes were then inductively organised under headings for reporting. Data extraction, coding and synthesis were carried out by a single reviewer. EPPI-Reviewer 4 software was used to manage data [11].

## Results

### Flow of literature through the review

**Fig. 1:**
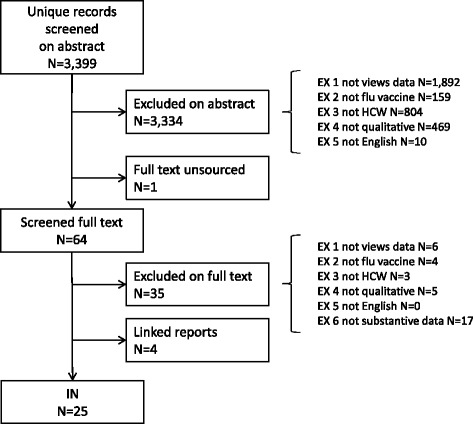
Flow of literature through the review

The flow of literature through the review process is shown in Fig. 1. A total of 3399 references were screened, which resulted in a total of 25 studies being included in the review, reported in 29 publications.

### Characteristics and quality of the studies

The results of quality assessment are shown in Additional file 2. The quality of the studies overall was mixed, with low scores particularly on the domains of sampling, ethics and bias, and transferability.

Tables 1 and 2 provide an overview of the studies. Table 1 shows those studies which collected data from HCWs about their own views of vaccination. Table 2 shows studies which included people delivering interventions, including infection control or occupational health staff, senior managers and administrators; some of these studies also included other stakeholders such as representatives of professional bodies, but none asked HCWs about their own views.

**Table 1:** Characteristics of the studies (HCWs)

Identifier	Participants	Sample size	Country	Setting
Clarke 2007 [12]	Patient care staff, administrators, directors, research staff, support staff	17	USA	Health department, private physician practices, Department of Human Services, university clinics, nursing home, schools, ambulance service
Hwang 2014 [13]	Doctors, nurses, pharmacy staff, patient service assistants, healthcare attendants	16	Singapore	Primary care
Isaacson 2009 [32]	Clinicians, nurses, medical assistants, support staff, office managers	32	USA	Primary care
Lehmann 2014 [14]	Doctors, nurses, students, other NS	123	Belgium, Germany, Netherlands	Hospital
Manuel 2002 [30]	Healthcare aides, nursing staff, dietary and maintenance staff	16	Canada	Nursing home
Nowak 2015 [15]	Physicians, nurses, allied health professionals, physician assistants	215	USA	NR
Pierrynowski Gallant 2007 [16, 43]	Nurses	11	Canada	Long-term care, mental health, acute care, public health
Prematunge 2014 [17]	Nurses, administrative/clerical staff, allied HCWs, research staff, technicians, facilities/logistics staff, physicians	3275	Canada	Hospital
Quinn 2014 [18]	Nurses	11	Ireland	Nursing home
Raftopoulos 2008 [19]	Nurses	30	Greece	Hospital, public health
Real 2013 [26]	Nurses, doctors, allied health staff	29	USA	Hospital
Rhudy 2010 [20]	Nurses	14	USA	Hospital
Seale 2016 [21]	Nurses, residents/registrars	41	Australia	Hospital
Seymour 2014 [22]	Public health staff including educators, outreach workers, nurses, dieticians, administrative staff	10	USA	Public health
Willis 2007 [23]	Nurses	71	USA	Hospital
Yassi 2010 [27]	Registered nurses, licensed practical nurses, unit clerks, physicians, care aides, dietary staff, housekeeping and kitchen staff, occupational therapists, librarians, hairdressers, laboratory staff, home support workers, psychiatric support workers, recreational aides	83	Canada	Long-term care, acute care, community care

**Table 2:** Characteristics of the studies (managers/implementers)

Identifier	Population	Sample size	Country	Setting
Hill 2015 [33, 44]	Nurses, physicians, infection control staff	7	USA	Spinal cord injury centre
Kalayil 2015 [45]	Infection prevention staff, occupational health staff	59	USA	Hospital
Khodyakov 2014 [28]	Employee health staff, infection prevention staff	26	USA	Hospital
Leask 2010 [24, 46]	Administrative leaders, clinician managers, Department of Health Staff, staff from universities, unions, professional groups	58	Australia	Hospital
Lim 2014 [31]	Immunisation directors, senior medical advisors, communicable disease directors, public health nurses	21	Australia	Hospital
Lindley 2014 [35]	NR	18	USA	Hospital, nursing home, community health services, home care services
Pianosi 2013 [29]	NR	21	Canada	University
Quach 2013 [25, 47]	Occupational health nurses, occupational health managers, infection control nurses	23	Canada	Acute care, continuing care, regional health authorities
Seale 2012 [34]	Infection control coordinators, clinical nurse consultants, nurse managers	29	Australia	Hospital

Most studies were carried out in the USA, Canada or Australia. The most commonly studied healthcare setting was hospitals or acute care facilities, followed by nursing homes or long-term care. Of the studies which looked at HCWs’ own views, eleven included a range of different HCW roles, while five focused specifically on nurses.

The thematic data were organised under the following headings for synthesis:

- beliefs about influenza, such as the risks and consequences of contracting influenza;
- beliefs about the vaccine, such as effectiveness and side-effects;
- ethical and organisational issues; and
- perceptions relating to interventions to promote vaccination.

### Beliefs about influenza

Many participants perceived themselves as at low risk of contracting influenza as they are healthy or ‘never get sick’ [12–25]. Some argued that they have a strong immune system due to working in healthcare and being exposed to infection [15, 18, 19, 23]. Several studies reported a perception that high-risk populations comprise older people and people with chronic illnesses, not healthy working-age adults: [14–16, 19, 20, 22, 25] *“what people get from the advertisements is you really only need it if you’re sick or in the nursing home or you have a lot of health issues”* (participant [25]). Few participants described themselves as at high risk, [16, 17, 19, 22] although some participants cited having chronic illnesses such as asthma as a reason to receive vaccination [14, 16, 17, 22].

In some studies, HCWs estimated that they were unlikely to transmit influenza to patients, [15, 16, 19, 20, 22] and a few suggested that patients are more likely to catch influenza from other patients than from HCWs [15, 16]. Many participants also thought that influenza is not serious, and should be easily manageable for healthy adults; [12, 13, 15, 16, 20, 22, 23] a few suggested that influenza is sometimes confused with the common cold [13, 16].

### Beliefs about vaccine

Many participants believed the vaccine was effective, and cited as reasons to accept vaccination: protecting patients against infection, particularly vulnerable groups such as older or immuno-compromised patients; [12, 14, 16–20, 22–24, 26–29] protecting their own health [12, 14–17, 19, 20, 22–24, 28, 29] or that of their families; [12–17, 22, 23, 28] and avoiding time off work [12, 14–18, 23, 24, 29]. Few participants mentioned herd immunity or population health [16, 17, 26]. Authors of several studies suggested that the balance of the data showed self-protection to be a more important motivator than the protection of patients [14, 15, 20, 22–24]. Several participants argued that vaccination is implied by the commitment to patient wellbeing which is a basic part of HCWs’ professional ethos: [12, 17, 26, 28] “*[I]t’s the Hippocratic Oath. The first thing you do is ‘do no harm’ and if you’re carrying around flu germs from patient to patient, you’re doing harm*” (participant [12]).

By contrast, other participants expressed a belief that the vaccine is not effective in preventing influenza [13–25, 30, 31]. Some participants pointed to the mutation of the virus and the possible mismatch of vaccine strains as reasons why the vaccine is sometimes ineffective [14, 15, 20, 23]. Some suggested that the scientific evidence on the effectiveness of the vaccine was insufficient to provide a convincing case for vaccination programmes: [18, 24, 31] “*I think it’s the skeptics that in actual fact are specifically the medical staff who are very analytical people, so therefore when you’re actually trying to actually use evidence as a means of mechanism of influencing people, I think that sometimes the evidence is a little rubbery*” (participant [31]). Several argued that other infection control procedures (such as handwashing and not working when ill) should suffice to prevent influenza without recourse to vaccination of HCWs [14, 19, 20, 22, 23].

Many participants expressed concern about possible side-effects of the vaccine, [12–25, 32, 33] including causing influenza or influenza-like symptoms, [13–18, 22–24] or discomfort at the injection site [13, 14, 16]. A few also mentioned more serious possible side-effects such as Guillain-Barré syndrome [12, 20, 25]. These views tended not to be based on evidence but on personal experience of adverse effects, [13–18, 20, 22–24] or in fewer cases, the experiences of colleagues [13, 22] or patients [18, 25]. Participants in two studies cited uncertainties about the scientific consensus on vaccine safety [16, 17]. However, one study indicated that staff implementing vaccination campaigns saw concern about side-effects as more motivated by media coverage or “gossip” between HCWs, which could create anti-vaccine cultures at department or unit level [25].

A few participants suggested that natural remedies or alternative therapies are more effective means of preventing disease than vaccination, [13, 16, 18, 19, 22] or expressed more specific anti-vaccine beliefs, for example arguing that vaccination can overload the immune system: [17, 22, 32] “*[Y]ou’re getting extra drugs in your system, and I do think things add up. [...] I just prefer a society that doesn’t think drugs, either to prevent or heal, before thinking of other ways*” (participant [22]).

### Ethical and organisational issues

Participants in several studies argued that as a matter of principle, the decision as to whether to accept vaccination is up to the individual HCW and should be respected [14, 17, 18, 20, 22, 23, 25, 27, 29]. There was some variation in how participants saw this ethical question. One participant identified a tension between the demands of patient protection, which are seen to point to mandatory vaccination, and the principle of autonomy: “*I have a real ethical problem with that. The nurse in me says it should be mandatory. But then the citizen in me says what happened to free choice? It’s a conflict”* (participant [25]). In another study, by contrast, participants suggested that autonomy is inseparable from HCWs’ professional norms and commitments: “*I feel it would take away our own decision-making really, and our own expertise, and that, you know, as nurses, part of our work ethic really is to advise other people and that surely we are able to make a decision for ourselves”* (participant [18]).

Participants in several studies mentioned management encouraging them to accept vaccination, [13, 14, 16, 17, 32, 34] and in some cases senior management ‘set an example’ by being vaccinated themselves [16, 17, 34]. However, in other cases participants felt that management were not really interested in promoting vaccination: [31, 32, 34] “*No, I think most of them aren’t doing enough and most of them think that they’re saving money by not getting everybody vaccinated*” (participant [31]). Participants also reported that peers and colleagues may have an influence, either in favour of vaccination [13, 14, 16–18, 29–31, 33] or against [13, 14, 22, 25, 27, 30].

Where management did actively promote vaccination, there was sometimes a perception that this was driven by an agenda of increasing productivity or promoting patient safety, and not for any concern for HCWs’ own wellbeing [18, 27, 30]. In some cases participants felt that institutional policies focused exclusively on increasing vaccination uptake as an end in itself, [27, 30] and that the resulting pressure on HCWs reflected a broader shift in the relation between management and staff, at the expense of the latter [18, 27].

### Interventions to promote vaccination

Several studies investigated participants’ preferences for information or education around vaccination. Participants reported a preference for messages which: are targeted to HCWs rather than generic messages aimed at the public; [12, 15, 18, 27] provide factual information and address specific concerns around vaccine effectiveness and risks; [12, 15, 16, 20–22, 27] and are based on robust evidence [12, 18, 27]. Some participants expressed frustration with existing educational programmes aimed at HCWs, finding them ‘dumbed down’ and insufficiently evidence-based [18, 21, 27].

Participants in five studies described experiences with declination form programmes in which HCWs who do not wish to be vaccinated are required to sign a form stating that they understand the consequences of this decision [22, 25, 28, 33, 34]. Perceptions of these programmes were mixed. Some participants who had been involved in implementing such interventions found them to be valuable both in directly shaping behaviour, and in providing opportunities to engage with HCWs and to shift norms around vaccination at an organisational level [28, 33]. Others thought the programmes had been ineffective, due to logistical challenges or resistance from HCWs, which led some organisations to dilute or abandon planned programmes [25, 34]. Some participants were also sceptical as to whether such programmes facilitate meaningful engagement with HCWs in reality: [25, 28] “*I was foolishly thinking that declination was going to make people think about how important [influenza vaccination] is, but it didn’t*” (participant [28]).

Several studies also investigated ‘hard’ mandatory policies, such as requiring HCWs to be vaccinated as a condition of employment, although these data were largely hypothetical and not based on participants’ direct experience (with one exception [35]). At least some implementers and managers were in favour of such policies, seeing them as the only way to get beyond the limitations of voluntary programmes: [24, 25, 34, 35] “*Until it’s mandatory, organizations flounder and we do the best we can with intimidation and prizes*” (participant [25]). However, some expressed doubt as to whether their organisation has the infrastructure and resources to enforce a mandate with sufficient stringency [25, 34]. Several participants also expressed concern about the ethics of mandatory programmes and the violation of HCWs’ autonomy [18, 20, 22, 25, 27]. Some believed that coercion would ultimately prove counter-productive by undermining respectful relationships between employers and employees: [25, 27] “*I think the coercion backfires in that it gets people’s backs up, and then they become more polarized*” (participant [27]).

## Discussion

This is the first systematic review to synthesise the qualitative literature on HCWs’ attitudes to influenza vaccination. Our findings support the conclusions of previous reviews of the quantitative literature that vaccination behaviour is complex and likely to be influenced by a wide range of determinants [36]. Our findings regarding individual perceptions of vaccination are broadly in line with what survey data have shown, particularly concern about side-effects and the importance of protecting oneself and one’s family, and also perceptions of low risk and seriousness [7, 36].

The qualitative literature suggests that many participants are sceptical about the value of vaccination programmes, but the sources of this vary. For some it derives from evidence-based arguments: these participants argue that the existing research literature does not provide sufficient robust evidence of benefit to patients to merit a wholesale change in policy. Others question the effectiveness or safety of vaccination on the basis of non-standard views about health more generally, as shown by the idea that vaccines ‘overload’ the immune system or that alternative therapies are preferable as a means of preventing disease. A subset of the qualitative studies also point to the importance of social and institutional factors, which have not been extensively explored in the quantitative literature. Some interventions are perceived as disempowering and as lacking in respect for HCWs’ professional judgement. This applies to coercive mandatory programmes, but also information campaigns which do not engage with what HCWs see as legitimate concerns about vaccination programmes. Such programmes are seen to disregard not just HCWs’ individual beliefs, but the professional norms and integrity which make it possible for healthcare organisations to function at all.

Views on the ethics of vaccination, and specifically of mandatory policies, appear to differ between the HCWs who are targeted by vaccination programmes and those who manage or implement them. The latter group take a largely individualistic view on the question as one of balancing abstract duties with individual rights. By contrast, some HCWs take a more social perspective which emphasises relationships – both their relation to their patients, and their employers’ relation to them. In this perspective the question is not whether the individual HCW has a right to refuse vaccination, but whether the organisation facilitates or hinders HCWs’ commitment to the care of their patients. This need not lead to questioning the value of vaccination, and in some cases it clearly acts as a motivator. Nonetheless, it seems to reflect a broader distinction between, in Gilligan’s terms, [37] an ethics of justice which emphasises abstract principles, and an ethics of care which emphasises interpersonal relationships. Programme implementers’ focus on an individualistic ethics of justice is largely in line with the assumptions made in the literature on the ethics of HCW vaccination, [38, 39] while the more social model implicit in HCWs’ views has received less attention. This suggests that in some cases HCWs’ resistance to vaccination campaigns may result from a different ethical perspective to that which has generally governed the design and implementation of these campaigns – not just from a different estimation of risks or benefits, or from misconceptions about the facts. Arguably this is borne out by the findings on the implementation of interventions, particularly education and declination form programmes: it appears that interventions which are well-grounded from the perspective of individual behaviour change sometimes face unexpected resistance from the social and organisational contexts within which they are received.

As noted above, we adopted a descriptive thematic synthesis approach partly for pragmatic reasons, and because the data were somewhat limited in their quality and depth, so a method like meta-ethnography might have been less suitable [40]. Other approaches such as ‘best fit’ framework synthesis would also have been possible, and could have enabled integration of other types of evidence in addition to qualitative studies [41].

There are some methodological limitations in the conduct and reporting of the primary studies, particularly around sampling strategies. The lack of clarity in available study reports as to how participants were sampled and recruited may limit the transferability of the findings. We did not exclude lower-quality studies from the review or downgrade them within the synthesis, and the potential limitations of the source data should be borne in mind when interpreting the review findings. We included only English-language studies, and almost all the included studies were conducted in North America or Australia, with only three from European countries; this may limit the generalisability of the findings to other contexts, particularly in relation to organisational culture. We did not include studies on pandemic influenza vaccination, although the findings of qualitative studies on this topic appear to be broadly similar to ours [42]. The body of evidence included in the review is not very extensive and many studies focus primarily on individual-level perceptions of vaccination; the findings on social and organisational factors discussed above are based on a fairly small number of studies. The evidence relating to interventions is skewed towards mandatory programmes, with few studies exploring voluntary strategies to promote vaccination, such as mobile vaccination carts or incentives.

## Conclusions

Many HCWs remain to be convinced of the seriousness of influenza, and of the effectiveness of vaccination programmes as a means of prevention. The organisational context – the pre-existing cultures and social networks which form the background to the implementation of policies or campaigns – may have an important influence on how interventions are perceived by HCWs. Intervention programmes would benefit from engaging with their target population to better understand their views and the process of decision-making about vaccination, and to situate these views in the context of the relationships between HCWs, management and patients.

## Additional files

Additional file 1: MEDLINE search strategy. (DOCX 14 kb)

Additional file 2: Results of quality assessment. (DOCX 18 kb)
