# Supplementary material for: Seasonal influenza vaccination of healthcare workers: systematic review of qualitative evidence
Source: BMC Health Serv Res. 2017 Nov 15;17:732. doi: 10.1186/s12913-017-2703-4 (PMC5688738; doi:10.1186/s12913-017-2703-4)
Supplement: Supplementary file 1 — MEDLINE search strategy. (DOCX 14 kb) [file 12913_2017_2703_MOESM1_ESM.docx]

**Additional file 1. Example search strategy**

Database: Epub Ahead of Print, In-Process & Other Non-Indexed Citations, Ovid MEDLINE(R) Daily and Ovid MEDLINE(R) <1946 to Present>

Search Strategy:

--------------------------------------------------------------------------------

1 exp health personnel/ or exp allied health personnel/ or exp dentists/ or exp nurses/ or exp nursing staff/ or exp personnel, hospital/ or exp pharmacists/ or exp physicians/ (419844)

2 ((healthcare or health or medical) adj2 (personnel or worker$ or staff or practitioner$)).ti,ab. (69914)

3 (doctor$ or clinician$ or physician$ or nurse$ or dentist$ or pharmacist$ or midwife$ or practitioner$).ti,ab. (878516)

4 1 or 2 or 3 (1152039)

5 Influenza Vaccines/ (18610)

6 Immunization/ (46785)

7 (vaccin$ adj4 (flu or influenza or SF)).ti,ab. (18076)

8 (immuni$ adj4 (flu or influenza or SF)).ti,ab. (3431)

9 5 or 6 or 7 or 8 (70432)

10 Influenza, Human/ (41367)

11 (flu or influenza).ti,ab. (83856)

12 10 or 11 (89997)

13 4 and 9 and 12 (3450)

14 exp animals/ not humans.sh. (4245677)

15 13 not 14 (3442)

16 Attitude/ (41988)

17 Attitude of Health Personnel/ (100062)

18 Health Knowledge, Attitudes Practice/ (83835)

19 Choice Behavior/ (25562)

20 Decision Making/ (75572)

21 16 or 17 or 18 or 19 or 20 (303606)

22 (attitude$ or barrier$ or belief$ or believ$ or choice$1 or choose$1 or consider$ or decision$ or experienc$ or facilitat$ or factor$ or influence$ or opinion$ or option$ or preference$ or promot$ or view or views or viewpoint$).ti,ab. (6772567)

23 21 or 22 (6882220)

24 Qualitative Research/ (27145)

25 qualitative.ti,ab. (152193)

26 "Surveys and Questionnaires"/ (344617)

27 (survey$ or questionnaire$).ti,ab. (759219)

28 24 or 25 or 26 or 27 (1023758)

29 23 or 28 (7307199)

30 15 and 29 (2287)
