# Supplementary material for: Seasonal influenza vaccination of healthcare workers: systematic review of qualitative evidence
Source: BMC Health Serv Res. 2017 Nov 15;17:732. doi: 10.1186/s12913-017-2703-4 (PMC5688738; doi:10.1186/s12913-017-2703-4)
Supplement: Supplementary file 2 — Results of quality assessment. (DOCX 18 kb) [file 12913_2017_2703_MOESM2_ESM.docx]

**Additional file 2. Results of quality assessment**

| **Study identifier** | **1. Abstract and title** | **2. Introduction and aims** | **3. Method and data** | **4. Sampling** | **5. Data analysis** | **6. Ethics and bias** | **7. Results** | **8. Transferability** | **9. Implications** |
| --- | --- | --- | --- | --- | --- | --- | --- | --- | --- |
| Clarke 2007 | Good | Good | Good | Poor | Fair | Fair | Good | Poor | Fair |
| Hill 2015 | Good | Good | Poor | Poor | Good | Poor | Good | Poor | Fair |
| Hwang 2014 | Fair | Poor | Fair | Poor | Poor | Fair | Fair | Poor | Fair |
| Isaacson 2009 | Good | Fair | Good | Fair | Fair | Poor | Poor | Fair | Fair |
| Kalayil 2015 | Good | Fair | Fair | Good | Fair | Poor | Fair | Fair | Fair |
| Khodyakov | Good | Fair | Fair | Good | Good | Poor | Fair | Fair | Poor |
| Leask 2010 | Good | Good | Fair | Fair | Fair | Good | Good | Fair | Fair |
| Lehmann 2014 | Good | Good | Fair | Fair | Poor | Fair | Good | Fair | Fair |
| Lim 2014 | Fair | Fair | Good | Fair | Fair | Poor | Fair | Poor | Fair |
| Lindley 2014 | Good | Fair | Poor | Good | Poor | Poor | Good | Fair | Poor |
| Manuel 2002 | Good | Fair | Poor | Poor | Fair | Very poor | Fair | Poor | Poor |
| Nowak 2015 | Good | Good | Poor | Poor | Very poor | Very poor | Fair | Poor | Fair |
| Pianosi 2013 | Fair | Fair | Fair | Fair | Fair | Poor | Poor | Poor | Poor |
| Pierrynowski Gallant 2007 | Good | Good | Fair | Fair | Good | Fair | Good | Fair | Good |
| Prematunge 2014 | Good | Fair | Good | Fair | Fair | Poor | Fair | Poor | Fair |
| Quach 2013 | Good | Fair | Fair | Fair | Good | Poor | Good | Poor | Fair |
| Quinn 2014 | Fair | Fair | Poor | Poor | Poor | Fair | Fair | Poor | Good |
| Raftopoulos 2008 | Fair | Fair | Good | Fair | Fair | Fair | Fair | Fair | Fair |
| Real 2013 | Fair | Fair | Poor | Very poor | Poor | Poor | Poor | Poor | Fair |
| Rhudy 2010 | Good | Good | Good | Fair | Good | Poor | Fair | Fair | Good |
| Seale 2012 | Good | Fair | Good | Good | Fair | Poor | Fair | Poor | Fair |
| Seale 2016 | Good | Fair | Good | Fair | Fair | Poor | Fair | Poor | Fair |
| Seymour 2014 | Good | Good | Good | Fair | Good | Fair | Good | Poor | Fair |
| Willis 2007 | Good | Fair | Good | Good | Fair | Very poor | Fair | Poor | Fair |
| Yassi 2010 | Fair | Fair | Fair | Poor | Fair | Very poor | Fair | Poor | Poor |
